# Comparison Between Primary and Secondary Pediatric Mucoepidermoid Carcinoma of the Head and Neck

**DOI:** 10.3389/fped.2020.00473

**Published:** 2020-08-20

**Authors:** Li Hao, Caixiao Shi, Ying Xu

**Affiliations:** Department of Otorhinolaryngology Head and Neck Surgery, The Affiliated Children Hospital of Zhengzhou University, Henan Children Hospital, Zhengzhou, China

**Keywords:** secondary malignant neoplasm, mucoepidermoid carcinoma, pediatric, salivary gland tumor, survival analysis

## Abstract

**Objectives:** Secondary mucoepidermoid carcinoma (MEC) of the head and neck is occasionally observed in childhood cancer survivors. The goal of this research was to compare the demographic and pathologic characteristics, as well as survival between primary and secondary MEC in children and adolescent patients.

**Methods:** Pediatric patients (younger than 19 years old) with surgically treated MEC of the head and neck were retrospectively enrolled at the Affiliated Children's Hospital of Zhengzhou University and divided into two groups based on their cancer history. Demographic, pathologic, and survival characteristics between the two groups were compared. The main study interests were recurrence-free survival (RFS), overall survival (OS), and disease-specific survival (DSS).

**Results:** The primary and secondary groups consisted of 63 and 15 patients, respectively. The two groups had similar distributions in terms of age, sex, tumor stage, neck lymph node stage, perineural invasion, lymphovascular invasion, p53, Bcl-2, proliferating cell nuclear antigen, carcinoembryonic antigen, and Ki-67 index. The 10-year RFS rates for the primary group and secondary group were 80 and 71%, respectively, and this difference was not significant (*p* = 0.464). The 10-year DSS rates for the primary group and secondary group were 83 and 82%, respectively, and this difference was also not significant (*p* = 0.649). The 10-year OS rates for the primary group and secondary group were 74 and 51%, respectively; this difference was significant (*p* = 0.023). Further Cox model analysis confirmed the independence of a previous cancer history (*p* = 0.043) in decreasing OS.

**Conclusions:** Pediatric patients with secondary MEC exhibit similar demographic, pathologic, and molecular characteristics as primary patients but worse OS. These findings indicate that special disease management approaches might be needed for secondary patients.

## Introduction

Acute leukemia is one of the most common malignancies in children and adolescents (1), and it significantly threatens the future constructors and successors of society at home and abroad. Fortunately, the prognosis has been greatly improved because of the advent of numerous aggressive multimodality therapies, and an increasing number of long-term cancer survivors are expected. Recent evidence has reported that nearly half of nonrelapse deaths among 5-year survivors are caused by secondary malignant neoplasms (SMNs) (2, 3), the most common explanation for cancer-caused death.

Although it is rare, some authors have described mucoepidermoid carcinoma (MEC) of the head and neck as being involved in SMNs (4–9). Whether these rare tumors could carry survival differences compared with primary MEC remains unknown, as the current evidence is limited to case reports, descriptive research, and an original study consisting of only 11 cases (3, 5–7, 10–14). Our hospital is one of the largest children's hospitals in China, covering ~30 million people younger than 19 years in Henan Province. Therefore, in the current study, we aimed to compare the demographic and pathologic characteristics, as well as survival between primary and secondary MEC in children and adolescent patients.

## Patients and Methods

### Patient Selection

Medical records of patients younger than 19 years and with surgically treated MEC of the head and neck were retrospectively reviewed from January 1990 to December 2019. Detailed demographic and pathologic information of the enrolled patients was obtained, and cases with recurrent disease at initial treatment or enough follow-up information were excluded. Data regarding age, sex, previous malignancy history, operation record, pathologic sections, immunohistochemical (IHC) analysis, and TNM stage based on the eighth AJCC classification, as well as follow-up data, were extracted and analyzed.

### Important Variable Definition

Previous cancer history referred to a history of hematological malignancies or osteosarcoma or other kinds of malignant tumors. The involved anatomic sites included the oral cavity, major and minor salivary gland, nasal cavity and paranasal sinuses, and pharynx. Pathologic sections of all included patients were re-reviewed by two head and neck pathologists with at least 10 years of experience who were double-blinded. Perineural invasion (PNI) was considered to be present if tumor cells were identified within the perineural space and/or nerve bundle; lymphovascular invasion (LVI) was positive if a tumor was noted within the lymphovascular channels (15, 16). Disease grade was defined according to the World health Organization classification as follows (17): intracystic component <20% (+2), neural invasion (+2), necrosis (+3), ≥4 mitoses/10 high-power field (+3), and anaplasia (+4). Scores were added and ranged from 0 to 14. A score of 0–4 represents a low-grade tumor, 5–6 represents an intermediate-grade tumor, and 7+ represents a high-grade tumor.

### IHC Analysis

Immunohistochemical results were analyzed as described previously (18, 19). The level p53 overexpression was determined as follows: –/+, <25% tumor staining; ++, 25–50% tumor staining; + + +, 50–75% tumor staining; and + + ++, more than 75% tumor staining. Tumors with levels of + + + and + + ++ were classified as having p53 positivity. Similar standards were used for Bcl-2, proliferating cell nuclear antigen (PCNA), and carcinoembryonic antigen (CEA). The Ki-67 score (0–100%) was calculated by the ratio of the number of immunostained nuclei to the total number of nuclei in tumor cells. The counting was performed in three randomly selected fields at 400× magnification.

### Surgical Proposal

In our department, systemic examinations including ultrasound and computed tomography or magnetic resonance imaging were routinely performed for every patient. Fine-needle aspiration was administered for select patients in whom a small lesion required differential diagnosis from normal lymph nodes. The operation types were partial parotidectomy (PP), superficial parotidectomy (SP), and total parotidectomy (TP), which were mainly based on the pathologic properties and surgeon's experience. The facial nerve was preserved in every patient. Neck dissection was generally performed for a cN+ neck, advanced tumor stage, or high-grade disease. Adjuvant radiotherapy was administered if there was a positive margin, pathologic cervical metastasis, advanced tumor stage, high-grade disease, or facial nerve invasion.

### Statistical Analysis

Enrolled patients were divided into the primary MEC group and secondary MEC group based on previous cancer history, but patients with MEC that was detected at initial diagnosis of hematological malignancies or others were included in the primary group. Student *t*-test was used to compare the continuous variables between the two groups, and the χ^2^ test was used to compare the categorical variables between the two groups. The main study interests were recurrence-free survival (RFS), overall survival (OS), and disease-specific survival (DSS). The survival time of RFS was calculated from the date of surgery to the date of first disease recurrence or the last follow-up. The survival time of OS was calculated from the date of surgery to the date of death of any cause or the last follow-up. The survival time of DSS was calculated from the date of surgery to the date of MEC-caused death or the last follow-up. The Cox proportional hazard model was used to determine the independent risk factors. All reported *p*-values were two-sided; *p* < 0.05 was considered to indicate a significant difference, and all statistical analyses were performed with SPSS 20.0.

## Results

### Demographic Data

A total of 78 patients (33 male and 45 female patients) with head and neck MEC were enrolled for analysis. The primary MEC group consisted of 63 patients, including 27 (42.9%) males and 36 (57.1%) females. The mean age was 16.4 years, and 10 (15.9%) patients were younger than 14 years. Primary tumor sites were characterized as the parotid gland in 45 (71.4%) cases, submandibular gland in 10 (15.9%) cases, and minor salivary gland in 8 (12.7%) cases.

The secondary MEC group consisted of 15 patients, including 6 (40.0%) males and 9 (60.0%) females. The mean age was 15.8 years, and no patients were younger than 14 years. Primary tumor sites were characterized as the parotid gland in 13 (86.7%) cases and the submandibular gland in 2 (13.3%) patients. The initial diagnosis of previous cancer was leukemia in all patients. All patients previously received alkylating-based and anthracycline-based chemotherapy. The mean latency between the initial hematological malignancy and the diagnosis of head and neck MEC was 8.8 years with a range from 4 to 10 years. The two groups had similar sex (*p* = 0.840), age (*p* = 0.195), and primary tumor site (*p* = 0.322) distributions, but patients with secondary MEC tended to be adolescents (Table 1).

**Table 1:** Comparison of demographic and pathologic variables between the primary and secondary groups.

**Variables**	**Primary (*n* = 63)**	**Secondary (*n* = 15)**	***p***
**Age (years)**			
<14	10 (15.9%)	0 (0.0%)	
≥14	53 (84.1%)	15 (100.0%)	0.195
**Gender**			
Male	27 (42.9%)	6 (40.0%)	
Female	36 (57.1%)	9 (60.0%)	0.840
**Primary tumor site**			
Parotid gland	45 (71.4%)	13 (86.7%)	
Submandibular gland	10 (15.9%)	2 (13.3%)	
Minor salivary gland	8 (12.7%)	0 (0.0%)	0.322
**Tumor stage**			
T1 + T2	43 (68.3%)	12 (80.0%)	
T3 + T4	20 (31.7%)	3 (20.0%)	0.532
**Cervical lymph node stage**			
N0	54 (85.7%)	12 (80.0%)	
N+	9 (14.3%)	3 (20.0%)	0.691
**Perineural invasion**			
Positive	8 (12.7%)	2 (13.3%)	
Negative	55 (87.3%)	13 (86.7%)	1.000
**Lymphovascular invasion**			
Positive	7 (11.1%)	2 (13.3%)	
Negative	56 (88.9%)	13 (86.7%)	1.000
**Tumor grade**			
High	10 (15.9%)	4 (26.7%)	
Intermediate	30 (47.6%)	5 (33.3%)	
Low	23 (36.5%)	6 (40.0%)	0.522
PCNA^*^			
Positive	10 (15.9%)	3 (20.0%)	
Negative	50 (79.4%)	8 (53.3%)	0.676
**p53**^**#**^			
Positive	11 (17.5%)	2 (13.3%)	
Negative	44 (69.8%)	9 (60.0%)	1.000
**Bcl-2**^****&****^			
Positive	9 (14.3%)	3 (20.0%)	
Negative	52 (82.5%)	12 (80.0%)	0.695
Ki-67^#^ (Mean)	17.5%	15.3%	0.753
**CEA**^**∧**^			
Positive	10(15.9%)	2 (13.3%)	
Negative	50 (79.4%)	12 (80.0%)	1.000

* *Status of PCNA in seven patients remained unknown*.

# *Status of p53 in 12 patients remained unknown*.

& *Status of Bcl-2 in two patients remained unknown*.

# *Status of Ki-67 in 13 patients remained unknown*.

∧ *Status of CEA in four patients remained unknown*.

### Surgical and Pathologic Characteristics

In the primary group, among the 45 patients with parotid MEC, PP, SP, and TP were performed in 15 (33.3%), 23 (51.1%), and 7 (15.6%) patients, respectively. Branches of the facial nerve were partially resected in five (11.1%) patients. Negative margins were achieved in all patients. Pathologic tumor stages were categorized as T1 for 14 (22.2%) patients, T2 for 29 (46.0%) patients, T3 for 12 (19.0%) patients, and T4 for 8 (12.7%) patients. Perineural invasion and LVI were present in eight (12.7%) and seven (11.1%) patients, respectively. Tumor grades were distributed as follows: low in 23 (36.5%) patients, intermediate in 30 (47.6%) patients, and high in 10 (15.9%) patients. Neck dissection was performed in 15 (23.8%, 15/63) patients, among whom 10 were classified as cN+, and five were classified as cN0. In the 10 cN+ patients, eight patients were reported to have pathologic metastasis. In the five cN0 patients, one patient was reported to have pathologic metastasis.

In the secondary group, among the 13 patients with parotid MEC, PP, SP, and TP were performed in five (38.5%), five (38.5%), and three (23.1%) patients, respectively. Branches of the facial nerve were partially resected in one (7.7%) patient. Negative margins were achieved in all patients. Pathologic tumor stages were categorized as T1 for six (40.0%) patients, T2 for six (40.0%) patients, T3 for two (13.3%) patients, and T4 for one (6.7%) patient. Perineural invasion and LVI were present in two (13.3%) and two (13.3%) patients, respectively. Tumor grades were distributed as follows: low in six (40.0%) patients, intermediate in five (33.3%) patients, and high in four (26.7%) patients. Neck dissection was performed in three (20.0%) cN+ patients, and three patients were reported to have pathologic metastasis.

The two groups had similar tumor stage (*p* = 0.532), PNI (*p* = 1.000), LVI (*p* = 1.000), cervical lymph node stage (*p* = 0.691), and disease grade (*p* = 0.522) distributions (Table 1).

### IHC Results

In the primary group, PCNA, p53, Bcl-2, and CEA positivity were detected in 10 (15.9%), 11 (17.5%), 9 (14.3%), and 10 (15.9%) patients, respectively, and the mean Ki-67 index was 17.5% (SD = 14.4%). In the secondary group, PCNA, p53, Bcl-2, and CEA positivity were detected in three (20.0%), two (13.3%), three (20.0%), and two (13.3%) patients, respectively, and the mean Ki-67 index was 15.3% (SD = 13.8%). The two groups had similar distributions of these factors (all *p* > 0.05) (Table 1).

### Survival Analysis

During our follow-up with a median time of 10.5 (range = 1.0–23.5) years, in the primary group, 10 patients received postoperative radiotherapy, and recurrence occurred in 12 patients: nine cases locoregionally and three cases distantly. Death occurred in 12 patients, among whom eight patients died of uncontrolled MEC, one patient died of another malignant tumor, and one patient died of cerebral edema (Table 2). In the secondary group, three patients received postoperative radiotherapy, locoregional recurrence occurred in two patients, and distant metastasis was noted in two patients. Death occurred in six patients, of whom two patients died of uncontrolled MEC, two patients died of other malignant tumors, and one patient died of serious infection (Table 2).

**Table 2:** Death cause for the 18 patients.

**Causes**	**Primary group (*n* = 12)**	**Secondary group (*n* = 6)**
Uncontrolled MEC	8 (66.7%)	2 (33.3%)
Other malignant tumor	1 (8.3%)	2 (33.3%)
Serious infection	1 (8.3%)	1 (16.7%)
Bleeding	–	1 (16.7%)
Cerebral edema	1 (8.3%)	–
Accident	1 (8.3%)	–

**Figure 1:**
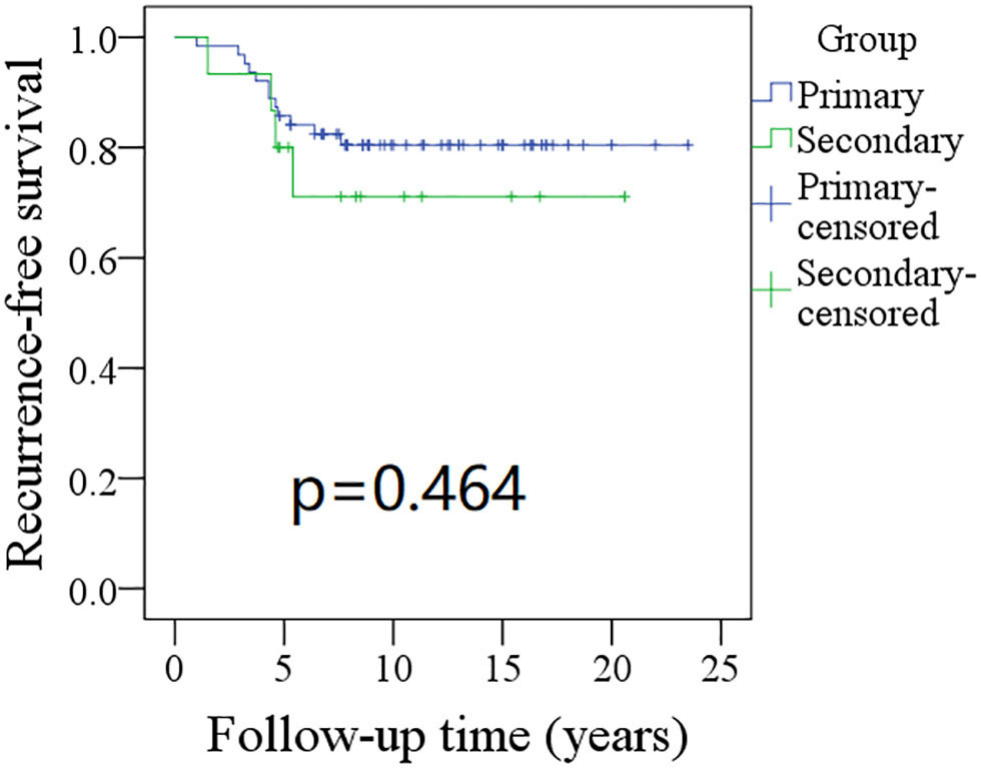
Comparison of recurrence-free survival (RFS) between primary and secondary groups: the 10-year RFS rates for the primary group and secondary group were 80 and 71%, respectively (*p* = 0.464).

**Figure 2:**
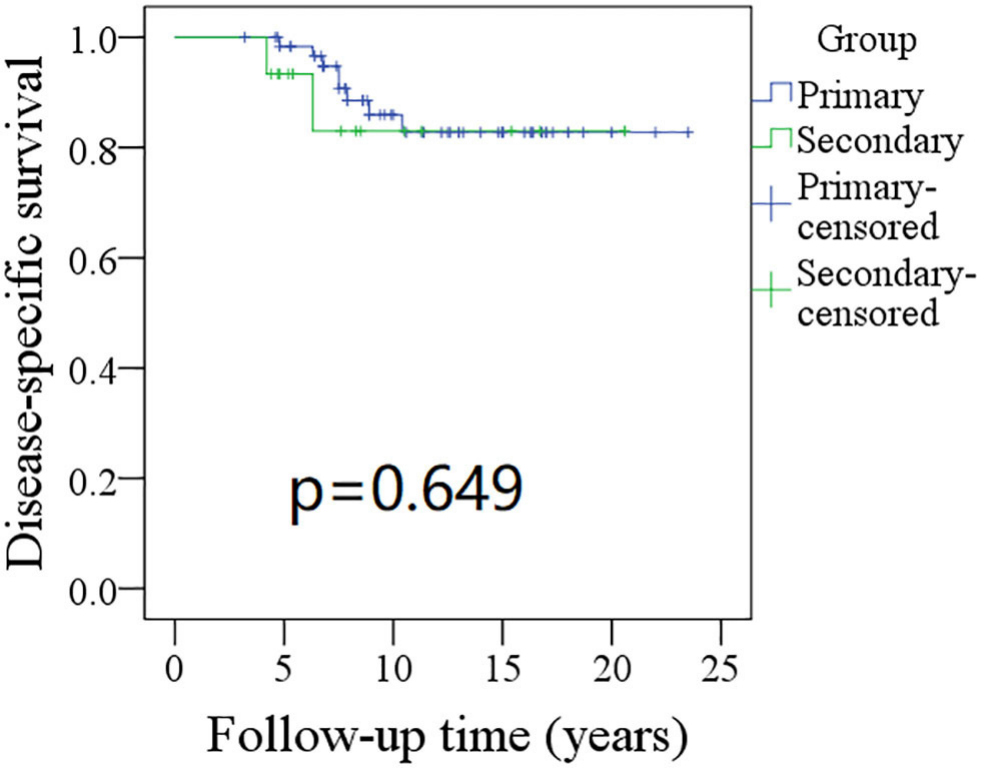
Comparison of disease-specific survival (DSS) between primary and secondary groups: the 10-year DSS rates for the primary group and secondary group were 83 and 82%, respectively (*p* = 0.649).

**Figure 3:**
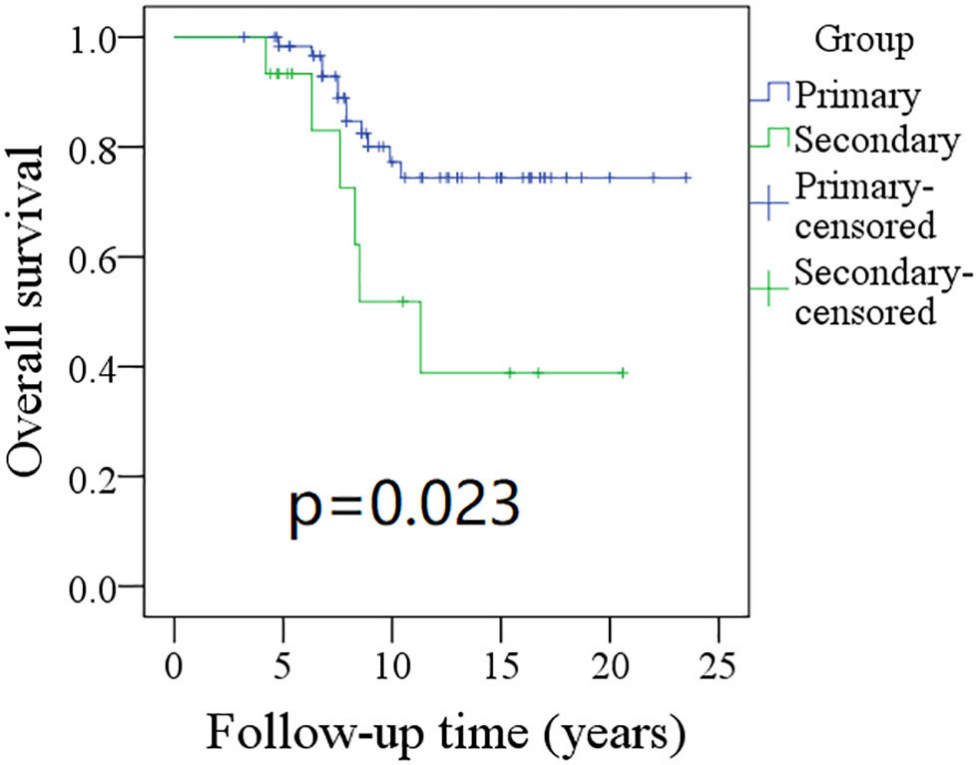
Comparison of overall survival (OS) between primary and secondary groups: the 10-year OS rates for the primary group and secondary group were 74 and 51%, respectively (*p* = 0.023).

The 10-year RFS rates for the primary group and secondary group were 80% and 71%, respectively, and this difference was not significant (Figure 1, *p* = 0.464). The 10-year DSS rates for the primary group and secondary group were 83 and 82%, respectively, and this difference was also not significant (Figure 2, *p* = 0.649). The 10-year OS rates for the primary group and secondary group were 74 and 51%, respectively; this difference was significant (Figure 3, *p* = 0.023). Further Cox model analysis confirmed the independence of a previous cancer history (*p* = 0.043) in decreasing OS (Table 3). Other independent prognostic factors included high tumor stage and cervical lymph node metastasis, which carried 3.231- and 4.227-fold risks for overall death, respectively.

**Table 3:** Univariate and Cox model analyses of overall survival in pediatric patients with head neck mucoepidermoid carcinoma.

**Variables**	**Univariate**	**Cox proportional hazard model**		
	**Log-rank test**	***p***	**HR**	**95% CI**
Age (<14 vs. ≥14 years)	0.336			
Gender	0.735			
**Primary tumor site**				
Parotid vs. others	0.185			
Cancer history	0.023	0.043	2.365	1.287–7.673
**Tumor stage**				
T1 + T2 vs. T3 + T4	0.017	0.033	3.231	1.456–10.825
**Neck lymph node stage**				
N0 vs. N+	0.005	0.017	4.227	2.004–13.715
Perineural invasion	0.371			
Lymphovascular invasion	0.219			
**Tumor grade**				
Low vs. others	0.003	0.086	3.816	0.956–8.462
Radiotherapy	0.541			

## Discussion

The most significant finding in the current study was that secondary head and neck MEC pediatric patients had similar demographic, pathologic, and molecular characteristics as primary MEC patients. The two groups had comparable RFS and DSS rates, but the secondary patients had lower OS than the primary patients, indicating that special disease management approaches may be needed for secondary patients.

The prevalence and risk factors for SMNs have been introduced by many researchers. Zakaria et al. (20) found that, among 22,635 people surviving childhood cancer, 395 patients had a secondary malignancy with an additional nearly fivefold risk than expected in the general population. The most common site was the thyroid, followed by the breast and oral cavity. The risk factors for developing SMN included being female, younger age at initial diagnosis of the primary cancer, and the primary cancer type. These authors also revealed that the standardized incidence ratios decreased with the time since diagnosis. Some conflicting results have been described by others; MacArthur et al. (21) reported that 55 secondary cancers occurred in 26,071 survivors of childhood and adolescent malignancy. The relative rate of a secondary malignant tumor was five times higher than expected in the general population. Absolute excess risk was noted in those diagnosed at an age younger than 10 years, but the authors concluded that the standardized incidence ratios were significantly elevated during the follow-up period. In another article by Chao et al. (22), the authors reported that cancer survivors had a nearly 2.5-fold higher risk for developing an SMN than age- and sex-matched populations. The risk factors comprised female sex, white ethnicity, advanced stage at first cancer diagnosis, and the use of radiotherapy. Similar findings were also reported by Henderson et al. (23), Scholz-Kreisel et al. (24), and Turcotte et al. (25). All these studies were descriptive but did not provide any data regarding MEC of the head and neck, possibly because of the rarity of this disease. Some authors have occasionally described MEC of the head and neck that occurred after pediatric sarcoma (6, 26), neuroblastoma (10), acute lymphoblastic leukemia (11, 12, 14, 26), or acute myelocytic leukemia (13). A recent study stated that salivary gland carcinoma accounted for 7.2% of 251 cases of SMN in pediatric leukemia survivors (3), but the authors did not further clarify the cancer type. In the current study, we also noted that all secondary patients had a previous leukemia diagnosis. These findings might be explained by alkylating agents, which are associated with an increased risk for SMN (9), as alkylating-based chemotherapy is usually an important part of leukemia treatment.

Demographic and pathologic characteristics of secondary MEC of the head and neck were extremely assessed systemically. Védrine et al. (7) might have been the first to find that, compared to patients with primary MEC, patients with secondary MEC had similar distributions in terms of age, sex, tumor grade, and tumor location. However, secondary survivors were more likely to have advanced clinical stage disease. However, there were only 18 cases included in the analysis in this study, and this small sample size might greatly decrease its persuasiveness. Recently, Verma et al. (27) reviewed 58 cases of secondary salivary MEC. These authors found that the parotid gland was most likely to be involved, that 87.5% of the diseases were low or intermediate grade, and that most of the patients were staged as T1 or T2. Similar findings were also noted in the current study; moreover, both PNI and LVI have been found to be important prognostic factors (28, 29). We are the first to report the rates of PNI and LVI in secondary patients, which were similar to those in primary patients. These findings are significant and can benefit the clinical management of this uncommon disease.

Survival of patients with SMN has been evaluated occasionally evaluated by previous studies. Keegan et al. (30) found that a previous cancer history was associated with a worse prognosis in patients with Hodgkin lymphoma, sarcoma, or breast, thyroid, or testicular cancers. A similar finding was also reported by Chao et al. (22); moreover, recently, Goldfarb et al. (31) noted that a longer latency time (>5 years) suggested an overall increased risk of death. However, none of these studies enrolled patients with head and neck MEC. In a previous study, Védrine et al. (7) reported that differences in OS, DSS, and RFS rates were not statistically significant between primary and secondary MEC groups; only 18 patients were included in their research. Verma et al. (27) reviewed 58 cases of secondary MEC in PubMed and reported that the 5-year RFS and OS rates were 95 and 93.4%, respectively, which were slightly better than our outcomes. One possible explanation was that there is much more bias in calculating the survival rates based on case reports worldwide. In a multicenter study by Seng et al. (32), the authors analyzed the effect of cancer history on RFS and DSS in MEC pediatric patients and revealed that a previous cancer history was significantly associated with decreased RFS and DSS. They proposed that there was a significantly adverse impact on the lymph defense system by previous chemotherapy for blood malignancy (32). However, note that the cervical metastasis rates for both groups were not more than 20% in the current study, which might aid in understanding our comparable results for RFS and DSS. To the best of our knowledge, this was the largest sample size study from a single medical center. Consistent with most previous studies (22, 31), our findings provide evidence to support that worse OS is more common in pediatric patients with salivary MEC if the patient has a previous cancer history. The importance of long-term surveillance in cancer survivors must be emphasized. Additionally, the significance of tumor stage and cervical nodal status has been widely analyzed. Both Fang et al. (29) and Seng et al. (32) reported that high tumor stage and neck lymph node metastasis were related to poorer survival, and our results also support this finding.

The limitations of the current study must be mentioned. There was inherent bias within the retrospective design, which would decrease our statistical power. Our sample size was still small, and there might be more interesting findings if a large sample size study was performed. Detailed data regarding the treatment of previous leukemia could not be obtained, and risk factors for secondary MEC could not be explored.

In summary, pediatric patients with secondary MEC exhibit similar demographic, pathologic, and molecular characteristics as primary patients but worse OS. These findings indicate that special disease management approaches may be needed for secondary patients.

## Data Availability Statement

All data generated or analyzed during this study are included in this published article. The primary data could be achieved from the corresponding author.

## Ethics Statement

The Zhengzhou University Institutional Research Committee approved our study, and all guardians of the patients signed informed consent agreements for medical research before the initial treatment. All methods were performed in accordance with the relevant guidelines and regulations.

## Author Contributions

All authors made the contribution in study design, manuscript writing, studies selecting, data analysis, study quality evaluating, and manuscript revising. All authors have read and approved the final manuscript.

## Conflict of Interest

The authors declare that the research was conducted in the absence of any commercial or financial relationships that could be construed as a potential conflict of interest.
